# Multi-sequence MRI based radiomics nomogram for prediction expression of programmed death ligand 1 in thymic epithelial tumor

**DOI:** 10.3389/fimmu.2025.1555530

**Published:** 2025-04-11

**Authors:** Jie Shen, Lantian Zhang, Shuke Li, Xiaofei Mu, Tongfu Yu, Wei Zhang, Yue Yu, Jing He, Wen Gao

**Affiliations:** ^1^ Department of Radiology, The First Affiliated Hospital of Nanjing Medical University, Nanjing, China; ^2^ Department of Oncology, The First Affiliated Hospital of Nanjing Medical University, Nanjing, China; ^3^ Department of Oncology, The Friendship Hospital of Ili Kazakh Autonomous Prefecture, Yining, China; ^4^ Department of Thoracic Surgery, the First Affiliated Hospital of Nanjing Medical University, Nanjing, China

**Keywords:** MRI, thymic epitelial tumors, radiomics, PD-L1, immunotharapy

## Abstract

**Background:**

High expression levels of programmed death receptor 1 (PD-1) and its ligand 1 (PD-L1) have been observed in thymic epithelial tumors (TET), suggesting their potential as prognostic indicators for disease progression and the effectiveness of immunotherapy in TET. The conventional method obtaining PD-L1 was challenging due to invasive sampling and tumor heterogeneity

**Methods:**

A total of 124 patients with pathologically confirmed TET (57 PD-L1 positive, 67 PD-L1 negative) were retrospectively enrolled and allocated into training and validation cohorts in a ratio of 7:3. Radiomics features were extracted from T1-weighted, T2-weighted fat suppression, and apparent diffusion coefficient (ADC) map images to establish a radiomics signature in the training cohort. Multivariate logistic regression analysis was conducted to develop a combined radiomics nomogram that incorporated clinical, conventional MR features, or ADC model for evaluation purposes. The performance of each model was compared using receiver operating characteristics analysis, while discrimination, calibration, and clinical efficiency of the combined radiomics nomogram were assessed.

**Results:**

The radiomics signature, consisting of four features, demonstrated a favorable ability to predict and differentiate between PD-L1 positive and negative TET patients. The combined radiomics nomogram, which incorporates the peri-cardial invasion sign, ADC value, WHO classification, and radiomics signature, showed excellent performance (training cohort: area under the curve [AUC] = 0.903; validation cohorts: AUC = 0.894). The calibration curve and decision curve analysis further confirmed the clinical usefulness of this combined model. The decision curve analysis demonstrated the clinical utility of the integrated radiomics nomogram.

**Conclusions:**

The radiomics signature serves as a valuable tool for predicting the PD-L1 status of TET patients. Furthermore, the integration of radiomics nomogram enhances the personalized prediction capability.

## Background

Thymic epithelial tumors (TET) are the most common anterior mediastinum neoplasms in adults (1, 2). Recently, programmed death receptor 1 (PD-1) and its ligand 1 (PD-L1) based immune checkpoint inhibitors (ICIs) show promising prospects in TET treatment. Additionally, PD-L1 has been proved as a predictor of the response to TET immunotherapy (2). TETs express PD-1/PD-L1 at high levels, which differs between different Masaoka stages and pathological subtypes (3–5). Furthermore, accumulating evidence has revealed high PD-1/PD-L1 expression in TET is worse prognosis (6). Therefore, accurate prediction of PD-L1 expression for TET patient is crucial. The common method to analysis PD-L1 expression is immunohistochemistry (IHC). However, IHC way is time consuming and does not provide a real-time detection (7). Thus, exploring a new approach to predict the expression of PD-L1 level for TETs may be clinical significance.

Radiomics methods have been widely applied with the extraction of numerous quantitative metrics on the entire tumor from radiological images including computed tomography (CT) and magnetic resonance image (MRI) (8, 9). Existing studies recognize the critical role of radiomics in reflecting tissue heterogeneity, staging and risk stratification of TET (10–12). Xiao et al. (10) using a combined radiomics nomogram incorporating tumor shape, apparent diffusion coefficient (ADC) value and radiomics signature differentiating different TET histologic subtypes. Mayoral et al. (11) applied radiomic features, conventional characteristics and both based on CT to differentiate thymoma and thymic carcinoma. A study compared 5 different models based on 13 representative features and a radiomics nomogram combining the selected clinical variables and radiomics signature to predict thymoma risk categorization (12). Besides, radiomics has been successfully employed to predict the level of PD-L1 in lung and esophageal tumors (13, 14). Zhang et al. (15) also reported that MRI radiomics could derive promising biomarker in discriminating PD-1/PD-L1 expression in intrahepatic cholangiocarcinoma.

In this study, we aimed to investigate the potential of multiparametric-MRI based radiomics model in evaluating the expression of PD-1/PD-L1 for TET patients.

## Methods

### Research design and patients involved

**Figure 1:**
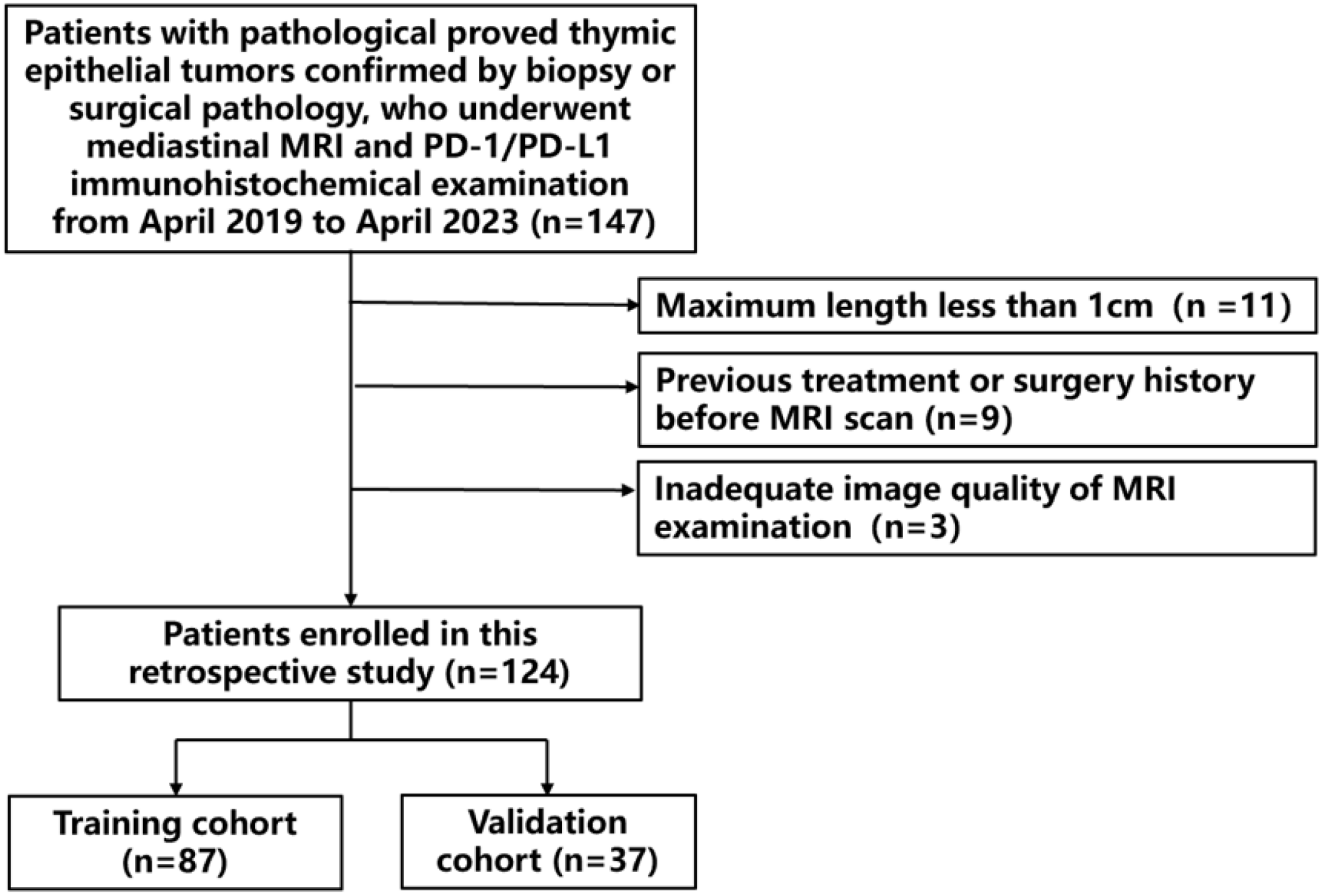
Flowchart depicting the process of patient enrollment and selection for analysis.

The ethical approval for this retrospective study was obtained from the Ethics Committee of our institute, and the requirement for written informed consent was waived. All radiological data and relevant clinical information were reviewed between February 2019 and March 2023. Patients included in this study met the following criteria: 1) confirmed pathological diagnosis of TET through CT-guided fine needle biopsy or surgery; 2) underwent mediastinum MRI with PD-L1 expression level determined by IHC test within one week interval. Patients with the following characteristics were excluded: 1) patients with a history of previous treatment for TET; 2) poor MRI image quality for further analysis. In total, a cohort of 124 TET patients with available PD-L1 results were enrolled in this study. The flowchart illustrating patient enrollment and analysis scheme is presented in **Figure 1**.

### PD-L1 expression status

PD-L1 expression levels were detected using the IHC method in this study. PD-L1 expression in tumors was determined by the PD-L1 22C3 pharmDx antibody and characterized by the Combined Positive Score (CPS), which is defined as the sum of PD-L1-positive cells (tumor cells and tumor-associated immune cells) per 100 tumor cells. The CPS cut-off values varied for different cancers, with a CPS of ≥1 defining positivity and a CPS of <1 defining negativity for PD-L1 expression (16). Two pathologists independently evaluated the PD-L1 expression status, with any inconsistent results being further analyzed by a senior pathologist.

### MRI data acquisition

All the MRI scans were performed using a 3.0 T MR scanner (MAGNETOM Skyra, Siemens Healthcare, Erlangen, Germany) with a 16-channel torso coil. The following sequences were used: 1) axial T1-weighted imaging with time of repetition [TR]/time of echo [TE], 140/2.5 ms; 2) axial T2-weighted imaging with fat suppression (TR/TE, 1200/93 ms). 3) axial diffusion weighted imaging (DWI) using an echo planar imaging (TR/TE, 4500/63 ms; *b* value, 0 and 1000 s/mm^2^), and corresponding ADC maps; 4) Axial dynamic contrast enhanced MR imaging was performed using a T1-weighed volumetric interpolated breath-hold examination with radial acquisition trajectory. Gadolinium-diethylene triamine pentacetic acid (Magnevist; Bayer Schering Pharma AG, Berlin, Germany) was intravenously bolus injected via a power injector at the rate of 4.0 mL/s at the dose of 0.1 mmol/kg, followed by a 20-mL bolus of saline administered at the same injection rate.

### Evaluation of conventional MRI features and ADC measurement

The conventional image features and ADC measurement were independently evaluated by two radiologists with 7 and 12 years of experience in chest radiology, respectively. In case of disagreements, a third senior chest radiologist with 30 years of experience was consulted to make the final decision. The evaluation of conventional features included: 1) presence or absence of cystic component; 2) presence or absence of internal septal; 3) presence or absence of pleural invasion; 4) presence or absence of pericardial invasion; and 5) measurement of ADC values. The evaluation of conventional MRI features and ADC measurements are shown in **Supplementary Method S1** and **S2**. All MR images were analyzed using a picture archiving and communication system (Vue PACS, version 12.1.5, Carestream Health, Rochester, NY).

### Segmentation, feature extraction, and selection

**Figure 2:**
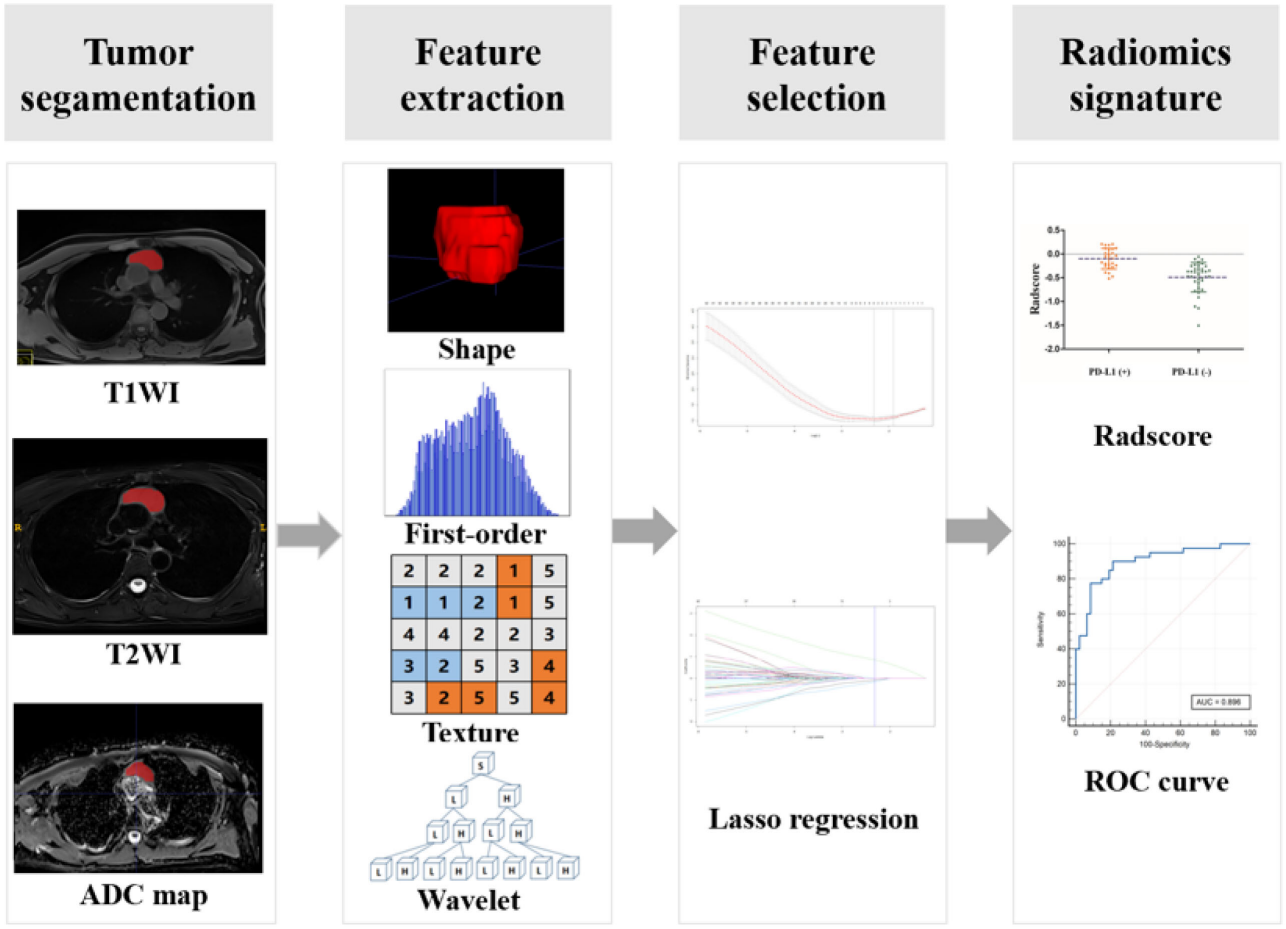
Workflow for the development of radiomics signatures.

The radiomics signature workflow is presented in **Figure 2**. The volume of interest (VOI) encompassing the tumor was manually delineated along the tumor contour on axial T2WI fat-suppression, axial T1WI without contrast enhanced, and ADC map images using ITK-SNAP software (version 4.0.0; University of Pennsylvania, Philadelphia, USA, http://www.itksnap.org/). This segmentation process included the cystic component of the tumor as well. Two experienced chest radiologists (with 7 and 11 years of experience respectively) completed the segmentation process. In case of any disagreement in determining the tumor mask, a final discussion was conducted to reach a consensus. Radiomics features were extracted from these ROIs using an in-house Python software (Pyradiomics version 2.12; http://pyradiomics.readthedocs.io/en/2.1.2/). A total of 788 features were extracted from T2WI fat-suppression, T1WI, and ADC map images individually. These radiomics features comprised four groups: Shape (n=14), first-order statistics (n=18), textural features (n=68), and wavelet features (n=688). The textural features consisted of Gray Level Run Length Matrix (GLRLM), Gray Level Size Zone Matrix (GLSZM), Gray Level Co-occurrence Matrix(GLCM), and Gray Level Dependence Matrix(GLDM) features. The detailed information regarding feature extraction is provided in **Supplementary Table S1**.

To ensure the stability of radiomics features extraction, a cohort of 20 TETs was randomly selected for a repeat segmentation. The first reader repeated the tumor segmentation after a week, while the second reader independently performed the segmentation to assess intra- and inter-class consistency.

All enrolled patients were randomly divided into a training cohort and a validation cohort at a ratio of 7:3. To address the variations in index dimensions of the data, the radiomics features extracted were normalized to standard normal distribution using z-scores. Consequently, a three-stage methodology was devised to reduce dimensionality and ascertain resilient attributes. The features selected for further analysis were those exhibiting high stability, as indicated by intra- and inter-class correlation coefficient values exceeding 0.80 (17). Furthermore, Pearson’s correction analysis was utilized to identify redundant and collinear features. Features exhibiting mutual correlation coefficients greater than 0.9 were subsequently excluded (18). In the third step of the analysis, we used the least absolute shrinkage and selection operator (LASSO) regression method to determine which features were the most closely associated with positive for PD-L1 expression. To ensure optimal performance, we employed 10-fold cross-validation for penalty parameter tuning. Thus, the resulting radiomics signature was created by combining these selected features in a linear fashion and weighting them according to their respective coefficients. This allowed us to calculate the corresponding Rad-score for each patient (18).

### Intra- and inter- observer concordance

To assess the stability of radiomics feature extraction, a cohort of 20 PD-L1 positive patients were randomly selected for repeat segmentation. Reader 1 performed tumor segmentation twice within one week, while Reader 2 independently conducted the segmentation to calculate intra- and inter-correlation coefficients (ICC).

### Selection of clinical features

We employed statistical tests (chi-squared for categorial variables and Wilcoxon for continuous variables) with a significant level of *p* < 0.05 to identify significant clinical features in the training group, as highlighted in **Table 1**. Subsequently, we performed univariate logistic regression analysis to assess the discriminatory ability of these features between two groups at a significance level of p < 0.05. Finally, employing backward stepwise multivariate logistic regression analysis, we identified independent predictive risk factors and constructed a predictive nomogram that incorporated both discriminative clinical features and Rad-score. Additionally, collinearity analysis using variance inflation factor (VIF) was conducted, leading to the removal of factors with VIF >10.

**Table 1:** Baseline characteristics between training and validation group of TET patients.

	Training Cohort (n=87)	Validation Cohort (n=37)	*P* value
Age (years)			0.322
Mean ± SD	54.84 ± 12.18	51.72 ± 13.87	
Gender			0.236
Male/Female	44/43	23/14	
Myasthenia Gravis			0.980
Present	12	6	
WHO classification			0.871
Low-grade	48	21	
High-grade and C	39	16	
PD-L1 level			0.997
High (CPS ≥ 1)	40	17	
Low (CPS <1)	47	20	

TET, thymic epithelial tumor; SD, standard deviation; C, carcinoma; PD-L1, programmed death receptor-ligand 1; CPS, Combined Positive Score.

### Model construction

The accuracy of the radiomics signature was initially evaluated in the training cohort using the area under the curve (AUC) of the receiver operating characteristic curve (ROC), and subsequently validated in an independent validation cohort. To enhance the clinical applicability of our model, we incorporated patients’ clinical characteristics and constructed a nomogram-based risk scoring system. Details regarding the calibration curve and decision curve analysis (DCA) of the combined radiomics nomogram are shown in **Supplementary Method S3**.

### Statistical analysis

The chi-square test or Fisher exact test was employed to compare categorical variables, as appropriate. For the analysis of continuous variables, either the student’s t-test or Mann-Whitney U test was utilized. Statistical analyses were performed using SPSS (version 22.0; IBM) and R software (Version 3.5.1; http://www.Rproject.org). All tests were two-tailed, and a significance level of p<0.05 was considered statistically significant. Univariable and multivariable logistic regression analyses were employed to identify significant risk factors. Following the multivariable analysis, the remaining variables were considered as potential risk factors and included in the clinical modeling of the training cohort. The DCA were employed to assess the clinical impact of the radiomics model, clinical model, and radiomics-clinical model in the training cohort using the “dca.R” package. The Delong non-parametric test was conducted to assess the statistical significance of the differences in AUC values among different models.

## Results

### Patient characteristics

The comparation for the differentiation of baseline characteristics of both the training and validation cohorts are presented in **Table 1**. The study population comprised 67 males and 57 females with a mean age of 53.1 ± 13.1 years (range, 26-80 years). Among these patients, 67 were negative and 57 were positive in PD-L1 expression. The detailed characteristics are shown in **Table 2**. The patients were randomly assigned to the training cohort and the validation cohort using computer-generated randomization.

**Table 2:** Comparison of conventional MRI features and radiomics score for TET patients of different PD-L1 expression levels.

	Training Cohort (n=87)			Validation Cohort(n=37)		
	PD-L1 (+) CPS ≥ 1, n=40	PD-L1 (-) CPS<1, n=47	*P* value	PD-L1 (+) CPS ≥ 1, n=17	PD-L1 (-) CPS<1, n=20	*P* value
Maximum diameter			0.203			0.117
Mean ± SD (cm)	4.87 ± 1.35	3.12 ± 1.89		4.52 ± 1.44	3.37 ± 1,25	
Cystic			0.226			0.138
Present	28	27		14	12	
Absent	12	20		3	8	
Internal septal			0.119			0.286
Present	18	29		9	14	
Absent	22	18		8	6	
Pleural effusion			0.256			0.478
Present	26	25		10	14	
Absent	14	22		7	6	
Pericardial effusion			0.001*			0.004*
Present	28	15		14	7	
Absent	12	32		3	13	
ADC value (×10^-3^ mm^2^/s)	0.890 ± 0.342	1.191 ± 0.357	0.001*	0.923 ± 0.285	1.276 ± 0.442	0.001*
Rad-score	0.73 ± 0.09	-0.40 ± 0.13	<0.001*	0.61 ± 0.04	-0.26 ± 0.15	0.019*

TET, thymic epithelial tumor; PD-L1, programmed death receptor-ligand 1; CPS, Combined Positive Score; SD, standard deviation; C, carcinoma; ADC, apparent diffusion coefficient, *P value < 0.05.

### Clinical, conventional MRI, and ADC features

Significant differences were observed in the prediction of PD-L1 expression in TET based on pericardial invasion sign, ADC value, and world health organization (WHO) pathological classification. The relationship between all these conventional features and PD-L1 expression was presented in **Table 2** for both the training and validation cohorts.

### Radiomics model performance

**Figure 3:**
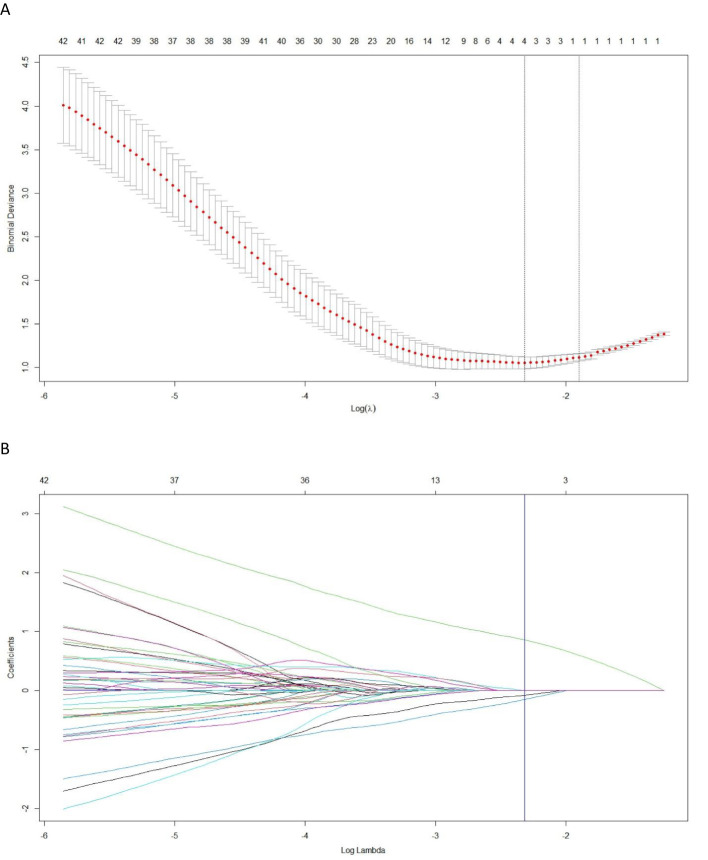
The least absolute shrinkage and selection operator (LASSO) regression is employed for the selection of radiomics features and construction of a signature. **(A)** In the LASSO model, the penalization parameter λ selection used 10-fold cross-validation as the minimum criteria. The log (λ) (x-axis) was plotted against the partial likelihood deviance (y-axis). Dotted vertical lines were drawn at the minimum criteria and the 1-SE criteria. λ value of 0.098, with log (λ), −2.317 was chosen (1-SE criteria). **(B)** LASSO coefficient profiles of the radiomics features. Ten-fold cross-validation in the log (λ) sequence was used to draw the vertical line at the value selected; also indicated are 4 features with nonzero coefficients.


Among the 2364 extracted radiomics features, a total of 2086 highly stable features (with inter- and intra-observer ICC values were 0.872 and 0.834, respectively) were selected for further analysis. After conducting pearson correlation analysis, an independent set of 4 features were identified. Subsequently, utilizing LASSO regression on the training cohort (**Figure 3**), four optimal features were chosen to construct the radiomics signature. The corresponding Rad-score for each patient was then calculated using the following formula:

$$\begin{array}{l} \text{Rad-score}=0.2166-0.00793*\text{A}\text{DC\_wavelet.HHL\_gIszm\_LargeAreaHighGrayLevelEmphasis}+\\ 0.0034*\text{T}1\text{WI\_log.sigma}.2.0.\text{mm}.3D\text{\_firstorder\_Minimum}+\\ 0.8575*\text{T}1\text{WI\_wavelet.LLH\_firstorder\_Minimum}-\\ 0.1540*\text{T}1\text{WI\_wavelet.HLL\_firstorder\_RootMeanSquared} \end{array}$$

### Radiomics-clinical combined models

**Figure 4:**
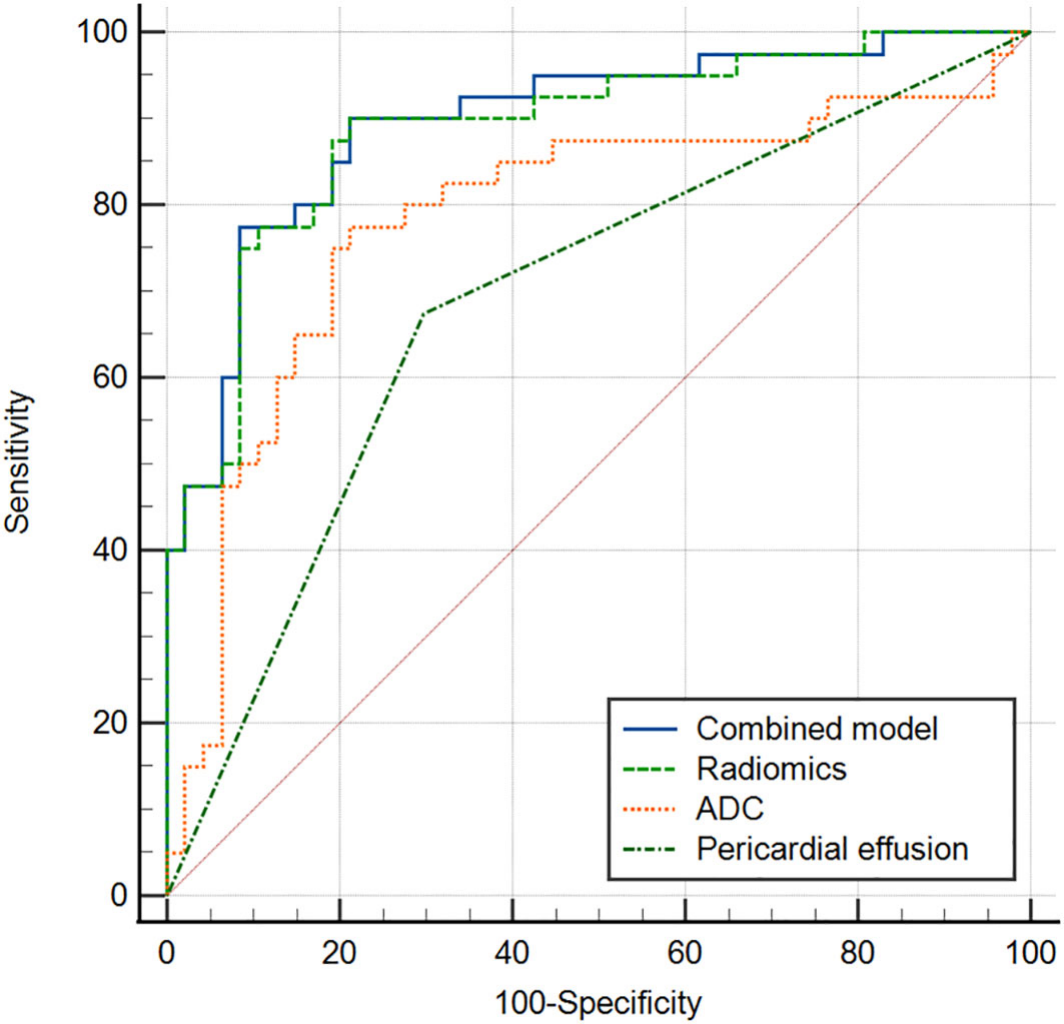
Nomogram of radiomics-clinical combined model for predicting PD_L1 expression level in TET.

**Figure 5:**
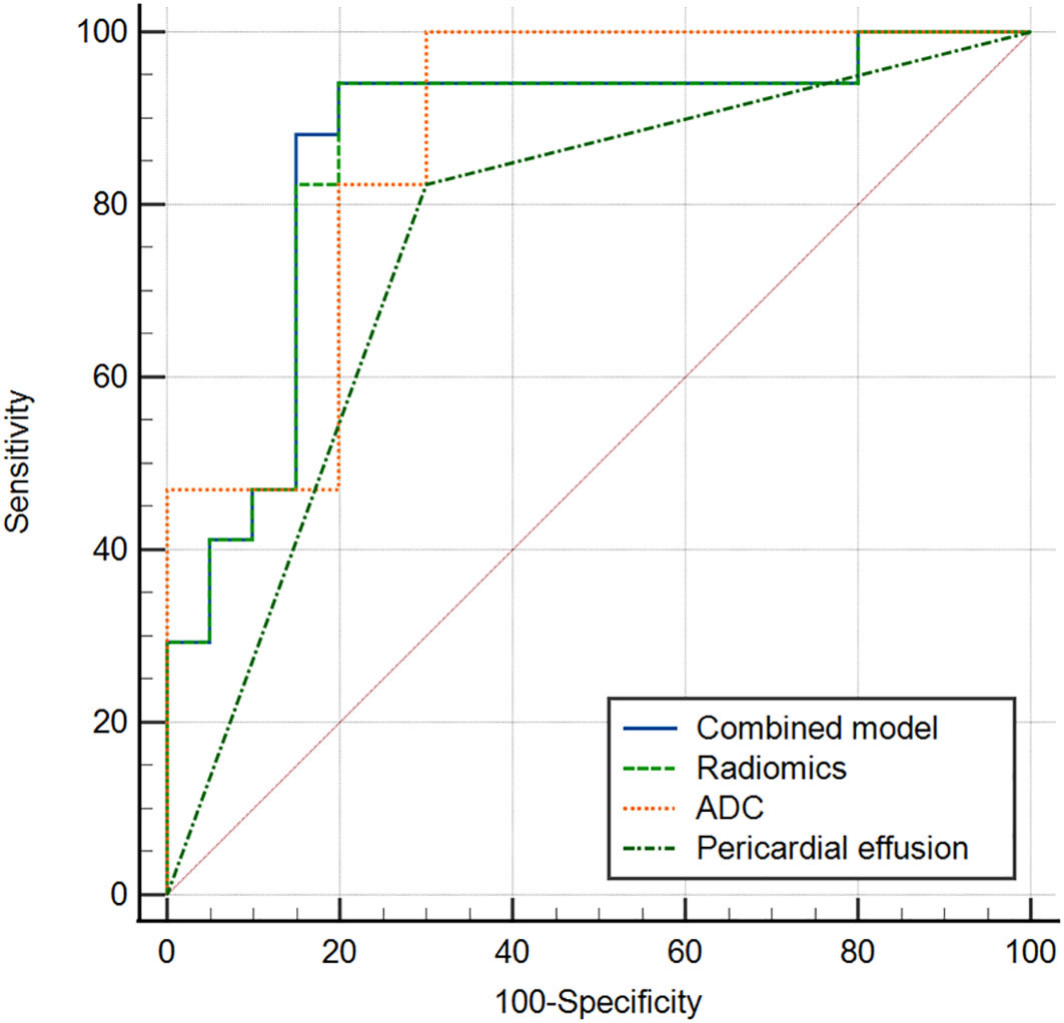
ROC curve of combined model, radiomics model, ADC and pericardial effusion sign for discriminating different PD_L1 expression (a training cohort, b validation cohort).

The multivariate logistic regression analysis revealed that the ADC value, pericardial effusion, and radiomics signature emerged as independent predictors of PD-L1 expression in the training cohort (**Table 3**). The combined model containing three indicators was shown in the nomogram (**Figure 4**). The discriminating efficiency of combined radiomics nomogram was confirmed in the ROC analysis with an AUC of 0.903 and 0.894 for the training and validation cohort, respectively (**Table 4**, **Figure 5**).

**Table 3:** Results of univariate and multivariate logistic regression for predicting PD-L1 expression in TET.

Variables	Univariate regression			Multivariate regression		
	OR	(95% CI)	*P* value	OR	(95% CI)	*P* value
Age	0.977	0.949-1.006	0.125	NA	NA	NA
Gender	1.408	0.594-3.337	0.437	NA	NA	NA
Myasthenia Gravis	1.761	0.599-5.178	0.304	NA	NA	NA
Maximum diameter	0.815	0.654-1.017	0.070	NA	NA	NA
Cystic component	1.995	0.748-5.318	0.167	NA	NA	NA
Internal septa	1.786	0.689-4.631	0.233	NA	NA	NA
Pleural effusion	2.941	1.061-8.143	0.226	NA	NA	NA
Pericardial effusion	6.103	2.407-11.474	<0.001*	9.706	1.210-17.863	0.032*
ADC value	0.704	0.592-0.837	<0.001*	0.769	0.613-0.965	0.023*
WHO classification	9.755	3.644-16.113	<0.001*	4.305	0.904-10.503	0.067
Rad-score	1.319	1.182-1.472	<0.001*	1.406	1.160-1.703	<0.001*

TET, thymic epithelial tumor; PD-L1, programmed death receptor-ligand 1; OR, odds ratio; CI, confidence interval; ADC, apparent diffusion coefficient, *P value < 0.05.

**Table 4:** The performance of radio_clinical combined model, radiomics model, ADC model and conventional MRI sign for the differentiating PD_L1 expression level in TET.

	AUC (95% CI)	Sensitivity (%)	Specificity (%)
Training Cohort (n = 87)			
Radio_Clinical Model	0.903 (0.821-0.956)	77.50	90.36
Radiomics model	0.889 (0.803-0.946)	89.60	78.72
ADC model	0.844 (0.751-0.913)	85.00	74.41
Pericardial effusion sign	0.712 (0.605-0.804)	70.00	72.34
Testing Cohort (n = 37)			
Radio_Clinical Model	0.894 (0.716-0.956)	82.35	85.00
Radiomics model	0.865 (0.712-0.955)	93.21	80.00
ADC model	0.816 (0.727-0.913)	89.71	70.30
Pericardial effusion sign	0.787 (0.593-0.886)	82.35	70.00

ADC, apparent diffusion coefficient; PD-L1, programmed death receptor-ligand 1; TET, thymic epithelial tumor; CI, confidence interval.

**Figure 6:**
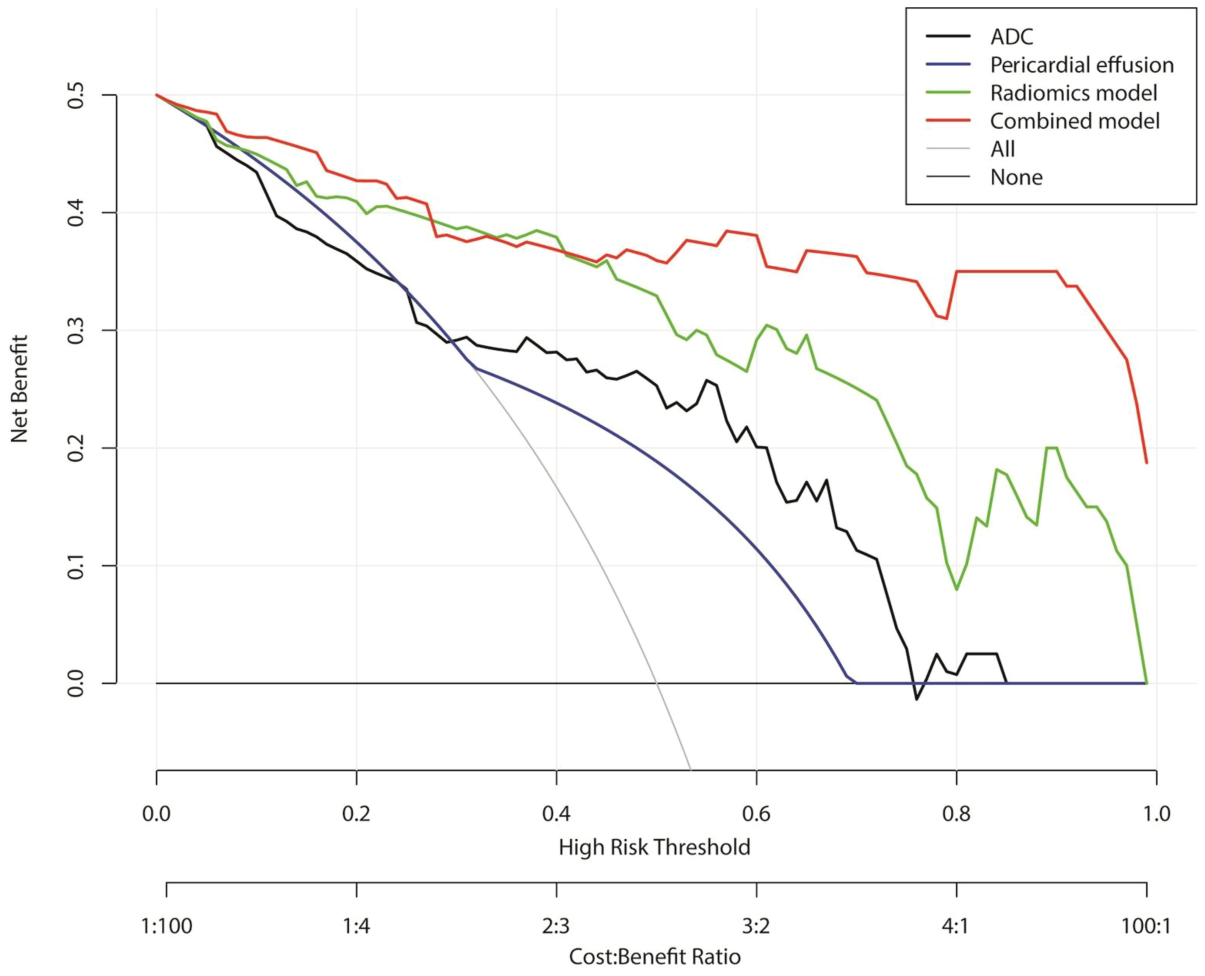
Decision curve analysis for conventional MRI, apparent diffusion coefficient, radiomics signature model, and combined radiomics nomogram for the combined training and validation cohort.

The results of the DCAs for conventional MR imaging, ADC model, radiomics signature model, and combined radiomics nomogram in both the combined and validation cohorts can be observed in **Figure 6**. The DCA curves demonstrate that the combined radiomics nomogram exhibits significant clinical usefulness when considering threshold probabilities exceeding 5%. This suggests that the combined radiomics nomogram serves as a dependable clinical tool for predicting PD-L1 status among TET patients.

## Discussion

Regarding immune checkpoints in TETs, considerable attention has been directed towards PD-1 and its ligand 1, eliciting escalating interest (2, 19). Previous studies have demonstrated a significant correlation between high PD-L1 IHC expression and enhanced response to pembrolizumab in patients with TET (20). A non-invasive, real-time monitoring approach for PD-L1 expression of TET capable of capturing spatio-temporal heterogeneity is imperative. In our study, the radiomics signature model based on multi-sequence MRI exhibited promising predictive performance in determining PD-L1 expression in TET. Furthermore, by integrating additional factors such as peri-cardial invasion sign, ADC value, and radiomics signature into a comprehensive nomogram, we observed further enhancement in the efficacy of the combined model. Consequently, we propose that utilizing a combined nomogram with radiomics could serve as an effective approach for predicting PD-L1 expression in TET.

In recent years, relevant studies on radiomics for immune therapy of tumor have been increasingly reported (7, 21, 22). Several studies have utilized radiomics methods to investigate the potential of predicting immune status in thoracic tumors (21, 23). Tian et al. (21) employed a CT based radiomics signature and predicted high PD-L1 expression of non-small cell lung cancer and to deduce clinical outcomes in response to immunotherapy. Zheng et al. (7) developed and validated a radiomic model based on contrast-enhanced computed tomography to predict the expression of PD-L1 in head and neck squamous cell carcinoma. Additionally, previous studies have demonstrated the robust performance of MRI-based radiomics biomarkers in predicting PD-L1 expression levels across various tumor type including hepatocellular carcinoma, cholangiocarcinoma and breast cancer (15, 23, 24). Specifically, our study was the first to employ a multi-sequence MRI radiomics integrated model for predicting the PD-L1 level in TETs. The observed variations in cut-off values could potentially be attributed to the diverse spectrum of cancer types and different imaging methods. In our study, we employed a CPS threshold of ≥ 1 as the cut-off value for determining PD-L1 positive expression in patients with TETs, aligning with treatment strategies established by previous studies (2, 25).

Radiomics has emerged as a burgeoning field that transforms medical images into an extensive array of high-dimensional imaging descriptors for oncological tissue (26). In our study, the radiological signature was constructed using a feature set consisting of four radiomics features, resulting in an AUC of 0.903 and 0.894 in the training and validation cohorts, respectively. Our model selected three distinct wavelet features and one traditional feature extracted from T1WI images and ADC maps. The wavelet features were extracted from the decomposed images, which undergo decomposition through the application of a wavelet transform filter. GLSZM (zone entropy and gray-level nonuniformity normalized) mainly describe patterns or the spatial distribution of voxel intensities. The first-order statistics features provide information related to the gray-level distribution of the image (7, 27). These features can comprehensively capture the essence of the original image and effectively extract intratumor heterogeneity information (28). Zhou et al. (18) have reported a radiomics signature based on MRI imaging incorporating 7 wavelet features in intrahepatic cholangiocarcinoma stratification, suggesting that wavelet transformation serves as a multiscale approach enabling comprehensive investigation of tumor biological characteristics and spatial heterogeneity.

According to the literature (29), a substantial proportion of tumors derived from patients with thymoma and thymic carcinoma exhibit expression of both PD-L1 and PD-1. Additionally, the PD-L1 positive group exhibited a higher proportion of high-grade TET patients compared to the negative group in our observation. In the previous study, Padda et al. (30) evaluated PD-L1 expression and also observed a positive correlation between higher PD-L1 expression levels and advanced WHO histologic grades (B2, B3, C), as well as an association with worse overall survival. Katsuya et al. (31) reported a significantly higher expression of PD-L1 in thymic carcinoma compared to thymoma. Finally, we developed a radiomics nomogram by integrating the radiomics signature, ADC value, and peri-cardial invasion sign, which exhibited improved calibration and discriminating efficiency in both training and validation cohorts. Several studies have demonstrated a positive correlation between higher PD-L1 expression and increased tumor aggressiveness in TET (25–32). Furthermore, a meta-analysis revealed significantly elevated levels of PD-L1 positivity in thymoma type B2/B3 or thymic carcinoma compared to thymoma types A/AB/B1 (5). This may elucidate the decreased ADC value and presence of pericardial invasion sign observed in conventional MRI of the PD-L1 positive group in TET.

Our study had some limitations and prospects. Firstly, our radiomics analysis was conducted on a single machine at a single institution. To provide high-level evidence for clinical practice, external validation in a larger sample from multiple institutions is necessary. Secondly, the cohort size, particularly the limited number of PD-L1 positive cases, was relatively small in this study. Thirdly, we only included a broad group of TET patients; however, future studies focusing on specific pathological subgroups of TET may yield more significant findings. Fourthly, this study focuses on the validation of PD-L1 prediction methodology. It mainly explores the feasibility of using MRI to predict PD-L1 expression and provides a non-invasive and rapid PD-L1 status evaluation tool for clinical practice to aid decision-making in precision diagnosis and treatment. In the subsequent prospective studies, we will include patients with high homogeneity and perform prognostic correlation analysis on the model.

## Conclusions

In our study, we have developed a radiomics signature based on multi-parametric MRI as a non-invasive and reliable method for predicting the expression level of PD-L1 in TET patients. Additionally, we have established a nomogram model that integrates the radiomics signature with clinical data, ADC values, and conventional MRI factors to enable personalized evaluation of PD-L1 expression levels in TET patients.

## Data Availability

The original contributions presented in the study are included in the article/**Supplementary Material**. Further inquiries can be directed to the corresponding author.
