# Supplementary material for: Multi-sequence MRI based radiomics nomogram for prediction expression of programmed death ligand 1 in thymic epithelial tumor
Source: Front Immunol. 2025 Apr 11;16:1555530. doi: 10.3389/fimmu.2025.1555530 (PMC12021882; doi:10.3389/fimmu.2025.1555530)
Supplement: Supplementary file 1 [file Table1.docx]

**Supplementary Method S1**

**The definition of conventional MRI features**

The maximal diameter was measured at the level where the tumor appeared largest. The tumor cystic component was classified as T2WI high signal without enhancement on the post-contrast T1WI sequence for estimating purposes. The presence of internal septal was defined as a linear or curvilinear structure that connects more than two slices in the tumor. Pleural or pericardial was characterized by low-signal intensity on T1W images and high-signal intensity on T2W images in the thoracic cavity or pericardial cavity.

**Supplementary Method S2**

**The measurement of apparent diffusion coefficient (ADC) value**

The apparent diffusion coefficient (ADC) measurements were followed as: three circular regions-of-interest (ROIs), approximately measuring about 0.5 cm^2^ each, were delineated on the solid tumor area that predominantly exhibited lower signal intensity on the ADC map. ROIs were carefully placed to exclude large fatty, necrotic, cystic, and hemorrhagic areas. The measured ADCs from these three ROIs were then averaged to obtain a mean ADC for further statistical analyses. The ADC value was calculated by fitting the b_0_ image and DW images at 1000 s/mm^2^ *b* value into the following equation: $S_{b}/S_{0}=exp\left( -b ADC \right)$. The final ADC value was the average of the ADC values of three ROIs.

**Supplementary Method S3**

**Calibration curve and decision curve analysis (DCA)**

The predictive performance of the combined radiomics nomogram was evaluated using calibration curves. These curves plot the nomogram-predicted probability of positive programmed death receptor ligand 1 (PD-L1) expression on the X-axis and the actual rate obtained through bootstrapping on the Y-axis. The agreement between the depicted calibration curve and a 45° straight line reflects the accuracy of prediction achieved by the combined radiomics nomogram.

To assess the clinical utility of the combined radiomics nomogram for individual prediction of PD-L1 status in TET, we applied DCA to the combined training and test cohort. The net benefit was plotted against the threshold probability. We constructed four decision curves based on pericardial effusion, ADC models, radiomics model, and combined radiomics nomogram respectively, demonstrating their clinical usefulness by calculating net benefits across a range of threshold probabilities.
