# Supplementary material for: Multi-sequence MRI based radiomics nomogram for prediction expression of programmed death ligand 1 in thymic epithelial tumor
Source: Front Immunol. 2025 Apr 11;16:1555530. doi: 10.3389/fimmu.2025.1555530 (PMC12021882; doi:10.3389/fimmu.2025.1555530)
Supplement: Supplementary file 2 [file Table2.docx]

**Supplementary Table S1:** Definitions of radiomics features extracted in this study.

| **Texture type** | **Texture name** |
| --- | --- |
| Shape features(n=14) | Elongation  Flatness  LeastAxisLength  MajorAxisLength  Maximum2DDiameterColumn Maximum2DDiameterRow  Maximum2DDiameterSlice Maximum3DDiameter  MeshVolume  MinorAxisLength  Sphericity  SurfaceArea  SurfaceVolumeRatio  VoxelVolume |
| First-order features (n=18) | 10Percentile  90Percentile  Energy  Entropy  InterquartileRange  Kurtosis  Maximum  MeanAbsoluteDeviation  Mean  Median  Minimum  Range  RobustMeanAbsoluteDeviation  RootMeanSquared  Skewness  TotalEnergy  Uniformity  Variance |
| Gray Level Co-occurrence Matrix (GLCM) features (n=22) | Autocorrelation  JointAverage  ClusterProminence  ClusterShade  ClusterTendency  Contrast  Correlation  DifferenceAverage  DifferenceEntropy  DifferenceVariance  JointEnergy  JointEntropy  Imc1  Imc2  Idm  Idmn  Id  Idn  InverseVariance  MaximumProbability  SumEntropy  SumSquares |
| Gray Level Run Length Matrix (GLRLM) features (n=16) | GrayLevelNonUniformity  GrayLevelNonUniformityNormalized  GrayLevelVariance  HighGrayLevelRunEmphasis  LongRunEmphasis  LongRunHighGrayLevelEmphasis  LongRunLowGrayLevelEmphasis  LowGrayLevelRunEmphasis  RunEntropy  RunLengthNonUniformity  RunLengthNonUniformityNormalized  RunPercentage  RunVariance  ShortRunEmphasis  ShortRunHighGrayLevelEmphasis  ShortRunLowGrayLevelEmphasis |
| Gray Level Size Zone Matrix (GLSZM) features (n=16) | GrayLevelNonUniformity  GrayLevelNonUniformityNormalized  GrayLevelVariance  HighGrayLevelZoneEmphasis  LargeAreaEmphasis  LargeAreaHighGrayLevelEmphasis  LargeAreaLowGrayLevelEmphasis  LowGrayLevelZoneEmphasis  SizeZoneNonUniformity  SizeZoneNonUniformityNormalized  SmallAreaEmphasis  SmallAreaHighGrayLevelEmphasis  SmallAreaLowGrayLevelEmphasis  ZoneEntropy  ZonePercentage  ZoneVariance |
| Gray Level Dependence Matrix (GLDM) Features  (n=14) | DependenceEntropy  DependenceNonUniformity  DependenceNonUniformityNormalized  DependenceVariance  GrayLevelNonUniformity  GrayLevelVariance  HighGrayLevelEmphasis  LargeDependenceEmphasis  LargeDependenceHighGrayLevelEmphasis  LargeDependenceLowGrayLevelEmphasis  LowGrayLevelEmphasis  SmallDependenceEmphasis  SmallDependenceHighGrayLevelEmphasis  SmallDependenceLowGrayLevelEmphasis |

A pool consisting of 788 radiomics features was developed. The radiomics features can grouped as follows: 1) shape (n= 14), first-order (n= 18), textural feature (n= 68) and wavelet features (n= 688).

Wavelet features:

Textural information was decoupled through discrete undecimated 3D wavelet transformation by separating the initial image, in which high-frequency signals were transformed to low-frequency signals. This is pre-processing before feature extraction. Specifically, with the original image X, and low-pass (L) and high-pass (H) wavelet functions, the wavelet decompositions of X were marked as X_LLL, X_LLH, X_LHL, X_LHH, X_HLL, X_HLH, X_HHL, and X_HHH. In this undecimated decomposition, each decomposed image was of equal size to the initial image, and all decompositions were shift-independent. Therefore, decompositions after wavelet transformation could be processed directly by initial description of the gross tumor volume. First-order feature X and textural features were extracted from each decomposition and resulted in 688 features.

The 14 shape features were extracted on original image; the 18 first order features and 68 textural were extracted on both original and the 8 filtered images. Therefore, the total number of radiomics features on each three-dimensional segmentation could be calculated as 14+ (18+68) ×9= 788.

Finally, 2364 features (which were extracted from the T1WI, T2WI and ADC map) were extracted from each patient.
